# Supplementary material for: Identification of potential biomarkers of vaccine inflammation in mice
Source: eLife. 2019 May 14;8:e46149. doi: 10.7554/eLife.46149 (PMC6555592; doi:10.7554/eLife.46149)
Supplement: Transparent reporting form [file elife-46149-transrepform.pdf]

eLIFE
1st Floor
24 Hills Road
Cambridge CB2 1JP, UK
P 01223 855340
W elifesciences.org
T @elife

eLife's transparent reporting form

We encourage authors to provide detailed information *within their submission* to facilitate the interpretation and replication of experiments. Authors can upload supporting documentation to indicate the use of appropriate reporting guidelines for health-related research (see EQUATOR Network), life science research (see the BioSharing Information Resource), or the ARRIVE guidelines for reporting work involving animal research. Where applicable, authors should refer to any relevant reporting standards documents in this form.

If you have any questions, please consult our Journal Policies and/or contact us: editorial@elifesciences.org.

Sample-size estimation

- You should state whether an appropriate sample size was computed when the study was being designed
- You should state the statistical method of sample size computation and any required assumptions
- If no explicit power analysis was used, you should describe how you decided what sample (replicate) size (number) to use

Please outline where this information can be found within the submission (e.g., sections or figure legends), or explain why this information doesn't apply to your submission:

This information can be found in *Materials and Methods* section *Animals, immunization and sampling*. A total of 840 samples have been collected from 8 groups of animals receiving vaccination or immune stimulatory treatment or an injection of saline solution. In each group, the following 7 time points were collected: 0, 4, 8, 24, 48, 72 and 168 hours. At each time point, 5 animals were sacrificed, and the injected muscle site, the iliac lymph nodes that drain the hind leg quadriceps muscles and peripheral blood were sampled. One sample (of group LPS immunization at 48h animal #5 peripheral blood sample) failed RNA labelling. Thus, transcriptomics data were obtained and analyzed from a total of 839 samples. The sample size was computed when the study was being designed. We used a power calculation ($n = 1 + 2C(s/d)^2$) where C is the constant (defined by the values of the probability ($\alpha = 0.05$) and the power ($1-\beta = 0.8$) = 7.85) and n = the group size required to fulfil the power requirement of the experiment, to determine the number of mice required to achieve a statistical power of 0.8 to reject the null hypothesis that there is no difference between the samples taken from the mice.

Replicates

- You should report how often each experiment was performed
- You should include a definition of biological versus technical replication
- The data obtained should be provided and sufficient information should be provided to indicate the number of independent biological and/or technical replicates
- If you encountered any outliers, you should describe how these were handled
- Criteria for exclusion/inclusion of data should be clearly stated
- High-throughput sequence data should be uploaded before submission, with a private link for reviewers provided (these are available from both GEO and ArrayExpress)

Please outline where this information can be found within the submission (e.g., sections or figure legends), or explain why this information doesn't apply to your submission:

Information about replicates can be found in *Materials and Methods* section *Animals, immunization and sampling - Total RNA preparation*.

Initial PCA and density plots for all samples were used to visually identify outliers. No outliers were identified. Internal control probes were removed.

As stated in *Materials and Methods* section *Whole-genome Microarray Analysis*, the complete set of the microarray data was deposited in NCBI's Gene Expression Omnibus and is accessible through GEO accession number GSE120661.

Statistical reporting

- Statistical analysis methods should be described and justified
- Raw data should be presented in figures whenever informative to do so (typically when N per group is less than 10)
- For each experiment, you should identify the statistical tests used, exact values of N, definitions of center, methods of multiple test correction, and dispersion and precision measures (e.g., mean, median, SD, SEM, confidence intervals; and, for the major substantive results, a measure of effect size (e.g., Pearson's r, Cohen's d)
- Report exact p-values wherever possible alongside the summary statistics and 95% confidence intervals. These should be reported for all key questions and not only when the p-value is less than 0.05.

Please outline where this information can be found within the submission (e.g., sections or figure legends), or explain why this information doesn't apply to your submission:

All statistical methods were described in *Materials and Methods*.

As stated in *Materials and Methods* section *Transcriptomic Analysis*, differential expression was evaluated using the moderated *t*-statistics and multiple test correction was done using Benjamini and Hochberg's (BH) method (Figure 1). Gene set enrichment analysis was performed using CERNO statistical test and the effect size was calculated using area under curve (AUC) (Figure 2).

Pearson correlation was used in Weighted gene correlation network analysis (WGCNA). WGCNA modules were compared to the blood transcriptional modules defined by Li *et al.* using hypergeometric test and *p*-values were adjusted for multiple testing using Benjamini and Hochberg's (BH) method (Figure 3).

Correlations of genes between tissues were calculated using the discordance/concordance score described by Domaszewska *et al* (Figure 4).

As stated in *Materials and Methods* section *Luminex and ELISA Analysis of Mouse Sera*, the significance of the change induced by different vaccines over those of saline alone were analysed using two-way ANOVA followed by Dunnett's multiple comparisons test (Figure 5, *p*-values that are significant after multiple testing were denoted with stars in (A) and exact *p*-values were shown in (B)).

Correlations between the fold changes of transcripts and proteins were calculated using Pearson correlation coefficient (Figure 6 & 7).

(For large datasets, or papers with a very large number of statistical tests, you may upload a single table file with tests, Ns, etc., with reference to sections in the manuscript.)

Group allocation

- Indicate how samples were allocated into experimental groups (in the case of clinical studies, please specify allocation to treatment method); if randomization was used, please also state if restricted randomization was applied
- Indicate if masking was used during group allocation, data collection and/or data analysis

Please outline where this information can be found within the submission (e.g., sections or figure legends), or explain why this information doesn't apply to your submission:

Experimental groups consisted of inbred mice which were housed randomly and treatment allocated.

Additional data files ("source data")

- We encourage you to upload relevant additional data files, such as numerical data that are represented as a graph in a figure, or as a summary table
- Where provided, these should be in the most useful format, and they can be uploaded as "Source data" files linked to a main figure or table
- Include model definition files including the full list of parameters used
- Include code used for data analysis (e.g., R, MatLab)
- Avoid stating that data files are "available upon request"

Please indicate the figures or tables for which source data files have been provided:

We developed an interactive web interface (available at https://vaccinebiomarkers.com) to facilitate data access and further discovery. This website allows users to (1) query genes and visualise their transcriptional profiles for each condition, (2) filter the differentially expressed genes by their functional groups and visualise the fold changes, and (3) analyse WGCNA modules. Code used for data analysis is available at https://vaccinebiomarkers.com.
